# Modulation of Membrane Lipid Composition and Homeostasis in Salmon Hepatocytes Exposed to Hypoxia and Perfluorooctane Sulfonamide, Given Singly or in Combination

**DOI:** 10.1371/journal.pone.0102485

**Published:** 2014-07-21

**Authors:** Marianne Olufsen, Maria V. Cangialosi, Augustine Arukwe

**Affiliations:** 1 Department of Biology, Norwegian University of Science and Technology (NTNU), Trondheim, Norway; 2 Department of Food and Environmental Science “Prof. G. Stagno d’Alcontres”, University of Messina, Messina, Italy; Bascom Palmer Eye Institute, University of Miami School of Medicine, United States of America

## Abstract

The relative importance of environmental hypoxia due to global climate change on organismal ability to adapt to chemical insult and/or mechanisms of these responses is not well understood. Therefore, we have studied the effects of combined exposure to perfluorooctane sulfonamide (PFOSA) and chemically induced hypoxia on membrane lipid profile and homeostasis. Primary salmon hepatocytes were exposed to PFOSA at 0, 25 and 50 µM singly or in combination with either cobalt chloride (CoCl_2_: 0 and 150 µM) or deferroxamine (DFO: 0 and 100 µM) for 24 and 48 h. CoCl_2_ and DFO were used to induce cellular hypoxia because these two chemicals have been commonly used in animal experiments for this purpose and have been shown to increase hypoxia-inducible factor 1-alpha (HIF-1α) and vascular endothelial growth factor (VEGF) levels. Fatty acid (FA) profiles were determined by GC-MS, while gene expression patterns were determined by quantitative PCR. Hypoxic condition was confirmed with time-related increases of HIF-1α mRNA levels in CoCl_2_ and DFO exposed cells. In general, significant alterations of genes involved in lipid homeostasis were predominantly observed after 48 h exposure. Gene expression analysis showed that biological responses related to peroxisome proliferation (peroxisome proliferator-activated receptors (PPARs) and acyl coenzyme A (ACOX)) and FA desaturation (Δ^5^- and Δ^6^-desaturases: FAD5 and FAD6, respectively) and elongation (FAE) were elevated slightly by single exposure (i.e. either PFOSA, CoCl_2_ or DFO exposure alone), and these responses were potentiated in combined exposure conditions. Principal component analysis (PCA) showed a clustering of peroxisome proliferation responses at transcript levels and FA desaturation against membrane FAs levels whose changes were explained by PFOSA and chemically induced hypoxia exposures. Overall, our data show that most of the observed responses were stronger in combined stressor exposure conditions, compared to individual stressor exposure. In general, our data show that hypoxia may, singly or in combination with PFOSA produce deleterious health, physiological and developmental consequences through the alteration of membrane lipid profile in organisms.

## Introduction

Anthropogenic activities leading to the emissions of carbon dioxide (CO_2_) and other greenhouse gases is thought to be the main contributor to climate change [1]. In the aquatic environment, effects of climate change have already been observed as increases in temperature and CO_2_ [2]. A consequence of increased water temperature is reduction in partial pressure of oxygen (pO2), and its availability to aquatic organisms [3], [4]. Oxygen is crucial for cellular respiration that generates energy for maintenance processes and development in aerobic organisms [5]. Thus, hypoxia (a quantifiable measure of climate change) may, singly or in combination with emerging pollutants such as perflourinated compounds (PFCs) produce deleterious physiological responses that may reduce general health conditions and impaired development in organisms [6].

Emerging compounds such as poly- and perfluoroalkyl substances (PFASs), organophosphate flame-retardants, detergent compounds, and several pharmaceutical substances have been linked to several biological effects in organisms and are continuously detected in the environment [7], [8]. PFASs are manufactured and used in various industrial and consumer products such as fluorinated polymers, surfactants, insecticides and aqueous fire-fighting foams [7]. In more than 50 years, 3M Company was the major producer of perfluorooctane sulfonyl fluoride (POSF) starting from 1949, but they have voluntarily phased out production in 2002 [9]. POSF is the precursor to several PFCs, whose reaction with methyl or ethylamides yields alkyl substituted sulfonamides: N-methyl perfluorooctane sulfonamide (NMeFOSA) and N-ethyl perfluorooctane sulfonamide (NEtFOSA), respectively. Further dealkylation can generate perfluorooctane sulfonamide (PFOSA), which is randomly distributed in biota and has been detected worldwide in fish, mammals, birds and humans at concentrations in the range of 1–100 ng/g wet weight of tissue [10], [11]. The chemical properties of PFOSA make the compound neither hydrophilic nor lipophilic and has been found to bind to carrier proteins, such as albumin, in blood [12]. PFASs can appear as both perfluorinated sulfonic (PFSAs) and carboxylic acids (PFCAs) which have been shown to exert a variety of biological effects, including – lipid homeostasis and peroxisome proliferation, hepatomegaly, immunotoxicity, uncoupling of mitochondrial oxidative phosphorylation, developmental toxicity, reduction of thyroid hormone circulation, necrosis, down-regulation of hepatic transporters and tumors [13], [14], [15], [16]. In mammalian systems, PFOSA was shown to undergo metabolic degradation at a slow rate to form PFOS, and can also undergo enterohepatic circulation, and mediate oxidative stress responses [17], [18].

Energy homeostasis and its regulation is critical for normal physiology and survival, and disruption of this balance often leads to chronic disease state [19]. FAs in fish tissues are present in different lipid classes and with different functions [20], [21]. There are two classes of essential long chain polyunsaturated fatty acids (PUFAs) omega-3 (n-3s) and omega-6 (n-6s), based on the location of the first double bond in the third (n-3) or sixth (n-6) position from the methyl end of the aliphatic carbon chain [22]. Conversions of these essential fatty acids (FAs) are orchestrated by several fatty acid desaturases (FADs) and elongase (FAE). Of the n-3 PUFAs, α-linolenic acid (ALA: 18∶3n-3) can be desaturated and elongated to form eicosapentaenoic acid (EPA: 20∶5n-3) through the activity of FAD6, FAE and FAD5, further transformation involves FAD4 and FAE to docosahexaenoic acid (DHA: 22∶6n-3) and is reversible. Whereas the n-6 PUFAs, linolelaidic acid (LA: 18∶2n-6) can be desaturated by FAD6 to γ-linolenic acid (GLA: 18∶3n-6) and elongated by FAE to dihomo-γ-linolenic acid (DGLA: 18∶3n-6) and further desaturation by FAD5 produces arachidonic acid (ARA: 20∶4n-6). ARA can thereafter through steps involving FAD4 and FAE transform into docosapentaenoic acid (DPA: 22∶5n-6), and this last step is reversible.

Chemically-mediated changes in the composition of lipids will affect many biological processes in the body, including lipogenesis, lipid transport, deposition and storage, peroxisome proliferation, and FA uptake in tissues and membrane fluidity [23]. Peroxisome proliferator-activated receptors (PPARs) are known to be critical regulators of lipid homeostasis by controlling the balance between burning and storage of long FAs [24]. PPARs are ligand-dependent transcription factors belonging to the nuclear hormone receptor superfamily [24]. The acyl coenzyme A (ACOX) catalyses the rate limiting-step in peroxisomal β-oxidation pathway of FA, and is commonly used as a biomarker for peroxisomal proliferation [24]. ACOX encoding gene in rats was regulated by PPARs through a peroxisome proliferator response element (PPRE) in the 5′ upstream region of the gene[24]. Regulation of peroxisome proliferation is controlled by PPARs and was first identified having this function in frogs (*Xenopus sp.*) [25]. They (PPARs) exert pleiotropic responses by regulating energy homeostasis, adipose tissue differentiation and maintenance, cell proliferation and tissue repair [26]. PPAR activities are consequently changed in accordance with a wide variety of physiological conditions, mediated through the ubiquitin-proteasome degradation system and extracellular signalling pathways and kinases that lead to receptor phosphorylation [27]. Administration of food containing PFOA induced peroxisome proliferation in Atlantic salmon (*Salmo salar*) [28]. In rats the same treatment has shown induced peroxisome proliferation and formation of benign liver tumors [16].

In this study, we have investigated biological pathways related to peroxisome proliferation, and lipid profile and homeostasis after exposure to chemically induced hypoxia and PFOSA, given singly and also in combination. Hypoxia was induced using cobalt chloride (CoCl_2_) and deferoxamine mesylate (DFO), two chemicals commonly used in animal experiments for this purpose and have been shown to increase hypoxia-inducible factor 1-alpha (HIF-1α) and vascular endothelial growth factor (VEGF) levels. DFO induces hypoxia by chelating iron for excretion and subsequently reducing the potential for oxygen transport [29] and CoCl_2_ is known to inhibit iron-dependent hydroxylases, resulting in an increase in HIF-1α protein accumulation, DNA binding activity, and transactivation function including VEGF induction [30], [31]. Given that optimal physiological condition is required for growth and development, optimal adaptation to hypoxic stress may have detrimental consequences resulting from inability to maintain physiological processes essential for normal cellular functions. It may also produce diminished capacity to handle fluctuation of other environmental factors that could ultimately lead to reduction in general fitness [32], [33], [34] and increase membrane (fluidity) passage for environmental contaminants. Our hypothesis is that exposure of salmon hepatocytes to hypoxia, singly or in combination with PFOSA, will produce significant changes in membrane lipid profile and biological processes that regulate membrane lipid homeostasis, with overt health, developmental, reproductive and physiological consequences.

## Materials and Methods

### Chemicals and reagents

Highly pure (>98%) linear perfluorooctane sulfonamide (PFOSA; CF_3_(CF_2_)_7_SO_2_NH_2_) isomer, as well as isotopically labeled linear PFOSA-^13^C_8_ and linear PFOS-^13^C_4_ were purchased from Wellington Laboratories (Guelph, ON, Canada). iScript cDNA Synthesis Kit and iTaq SYBR Green Supermix with ROX were supplied by BioRad Laboratories (Hercules, CA, USA). The original TA Cloning Kit PCR 2.1 vector, INVαF’ cells, TRIzol and Dulbecco’s Modified Eagle Medium (DMEM) with non-essential amino acid and without phenol red, fetal bovine serum (FBS), 0.4% trypan blue and L-glutamine were purchased from Gibco-Invitrogen Life Technologies (Carlsbad, CA, USA). Dimethyl sulfoxide (DMSO), penicillin-streptomycin-neomycin solution, collagenase (C0130-1G), bovine serum albumin (BSA), N-[2-Hydroxyethyl]piperazine-N’-[2-Ethane Sulfonic Acid] (HEPES), ethylenediaminetetraacetic acid disodium salt dihydrate (EDTA), ethyleneglycol bis-(β-aminoethylether)-N,N,N’,N’-tetraacetic acid (EGTA), polyunsaturated fatty acid 1 and 2 (PUFA1 and PUFA2) were purchased from Sigma-Aldrich Chemie GmbH (Munich, Germany). Tricaine methane sulphonate (MS-222) was purchased from Norsk Medisinaldepot AS. GelRed Nucleic Acid Gel Stain was purchased from Biothium (Hayward, CA, USA). The ZR Plasmid Miniprep-Classic was purchased from Zymo Research (Orange, CA, USA).

### Animals, exposure and sampling

All necessary permits were obtained from the Norwegian Animal Research Authority for the described study, which complied with all relevant regulations. Atlantic salmon (*Salmo salar*) were purchased from Lundamo Hatcheries (hatch and rearing centre located at Lundamo). Fish were kept at the animal-holding facilities for Department of Biology (Sealab, NTNU) in 100-liter tanks with continuously running fresh water at 10°C and flow rate of 40 L/h and natural photoperiod. Fish were acclimatized for two weeks and starved three days prior to liver perfusion.

### Collagenase perfusion, isolation and culture of hepatocytes

Prior to liver perfusion, all glassware and instruments were autoclaved and solutions were filtration sterilized by using 0.22 µm Millipore filter (Millipore AS, Oslo, Norway). Fish were anesthetised using MS-222 (70 g/L) administered 15 minutes prior to perfusion and euthanized after in accordance with regulations for animal research and approved by Norwegian Food Safety Authority (FOTS). Hepatocytes were isolated from 10 individuals by a two-step perfusion technique with modifications as previously described [35]. The cell suspension was filtered through a 150 µM nylon monofilament filter and centrifuged at 70×g for 5 min. Hepatocyte from individual fish were used across all individual exposure scenarios in such a way that all 10 fish were represented in all exposures. Cells were washed three times with serum-containing medium and finally resuspended in complete medium. Following collagenase perfusion and isolation of hepatocytes, viability of cells was determined by the trypan blue exclusion method. A cell viability value of >90% was a criterion for further use of the cells. Cells were plated on 35 mm TPP Tissue Culture Plates (Techno Plastic Products AG, Switzerland) at monolayer density of 2.1×10^6^ cells in 3 ml DMEM medium (without phenol red) containing 0.5% (v/v) FBS, 1% (v/v) L-glutamine, 15 mM HEPES and 1% (v/v) antibiotic-antimycotic.

### Plating of cells and exposure

Medium was added to plate prior to the cells, avoiding sedimentation of cells by rotating the tube every second plate. Cells were cultured at 10°C in a sterile incubator for 24 hours prior to exposure. After 24 hours pre-culture, growth medium was removed and quickly replaced with exposure medium (twenty wells for each exposure group); to 0.1% DMSO (control), 150 µM CoCl_2_, 100 µM DFO, 25 µM PFOSA (singly and in combination with either 150 µM CoCl_2_ or 100 µM DFO), 50 µM PFOSA (singly and in combination with either 150 µM CoCl_2_ or 100 µM DFO). This gave a total of 9 different exposure groups. Media and cells were harvested separately, ten wells for each exposure group at 24 and 48 h, post-exposure and snap-frozen immediately in liquid nitrogen. Cells used for RNA analysis were lysed in Trizol reagent for total RNA isolation according to the manufacturer’s protocol (Invitrogen).

### Assessment of cell viability

A pilot study using different concentration (10, 50, 100, 150 and 200 µM) of CoCl_2_ or DFO was performed in order to determine optimal exposure concentrations for hypoxia-inducing chemicals. Evaluation was performed using resazurin assay on cells exposed for 24 and 48 h in 96-well plates (2.1×10^5^ cells in 300 µl). After addition of rezasurin solution (10% of medium volume), cells were incubated for 6h at 10°C on a gyratory shaker. Samples were measured spectrophotometrically at 600 nm every 20 minutes. Viability was also investigated for all exposure groups (see below).

### Quantitative (real-time) PCR

Total cDNA for quantitative real-time polymerase chain reaction (q-PCR) analysis was generated from 1 µg total RNA from all samples using a combination of poly-T and random primers from iScript cDNA synthesis kit as described by the manufacturer (Bio-rad). RNA samples were evaluated for integrity using agarose gel electrophoresis. Quantitative real-time PCR was used for evaluating gene expression profiles for HIF1-α, FAD5, FAD6, FAE, ACOX and PPAR (α, β and γ). For each treatment, expression of individual gene targets was analyzed using the Mx3000P REAL-TIME PCR SYSTEM (Stratagene, La Jolla, CA, USA). Each 25 µl qPCR reaction contained - 12.5 µl of iTAQ SYBR Green Supermix with ROX (Bio-Rad), 1 µl of cDNA, 200 nM of each forward and reverse primers and remaining volume was autoclaved MQ-H_2_O. The three-step real-time PCR program included an enzyme activation step at 95°C (5 min) and 40 cycles of 95°C (30 s), 55–65°C (30 s) (depending on the primers used; see Table 1), and 72°C (30 s). Controls lacking a cDNA template were included to determine the specificity of target cDNA amplification. Cycle threshold (Ct) values obtained were converted into mRNA copy number using standard plots of Ct-value versus log copy number. The criterion for using the standard curve is based on equal amplification efficiency (usually 90%) with unknown samples and this is checked prior to extrapolating unknown samples to the standard curve. The standard plots were generated for each target sequence using known amounts of plasmid containing the amplicon of interest, as described previously by [36]. Data from each group were averaged and expressed as percentage of control.

**Table 1:** Primer pair sequences, accession numbers, amplicon size and annealing temperature conditions for genes of interest used for real-time PCR.

Target Gene	Primer sequence*		Amplicon size (basepairs)	Annealing temperature (°C)
	Forward	Reverse		
Hif-1α	GCT CAG AAA GTC GGT TGT CC	GCC AGC TCG TAG AAC ACC TC	152	60
FAD5	GAC CTA TAT TTC CAG CAT TAT CC	TCA CTC ATC TAC AAA TAG TAT TCC	192	55
FAD6	CAT CTG ATT CTG ATT CCA TTC C	CTC TGC TCC ACT CAC ACC	127	55
FAE	GAC ACC CAC GGA AAC CAT TAC	CTC TCC TAG CGA CAT TAC ATA CAG	111	55
PPARα	GCT TCA TCA CCA GGG AGT TT	TCA CTG TCA TCC AGC TCC AG	113	60
PPARβ	CAA TGG CTC GGA TCT CAA AT	ACT CTA CTG GGC TGG AGC TG	124	60
PPARγ	CAC TGT GAT CTG CAC TGT	ATG GCA TCA TGT GAC ATT	100	60

*Sequences are given in the 5′−3′order

### FA extraction and GC-MS analysis

Lipids were extracted from Atlantic salmon hepatocytes by homogenization in chloroform: methanol (2∶1) solution, added with 0.01% of 2,6-di-tert-butyl-4-methylphenol (BHT) as an antioxidant, according to the method of Folch et al [37]. FA methyl esters (FAMEs) from total lipids were prepared by acid-catalyzed transmethylation for 1 h at 100°C, using tricosanoic acid (23∶0) as internal standard. Methyl esters were extracted by c-hexane, then dried by centrivap, weighed and suspended in c-hexane (1% v/v). FAMEs analysis was performed using a Shimadzu GC-MS 2010 gas chromatograph-mass spectrometer and fitted with a fused silica capillary column (Supelco, Germany) and helium was used as carrier gas. The injector, detector and column temperatures were 250°C, 300°C and 200°C, respectively. Relative percentage of the area was obtained by using the following equation: Area% FAX = [AX/AR]×100, where: FAX = fatty acid to be quantified, AX  =  area of the methyl esters, X and AR = total area of the chromatogram. Peak areas lower than 0.1% of the total area was not considered. We identified FA methyl esters by comparing retention time of samples and standards.

### Statistics

Data are presented as mean percent of control with the same exposure duration ± standard error of mean (SEM). Normal distribution was assessed using Shapiro-Wilks test and homogeneity of variance was tested with Levene’s test. Comparison of different concentrations of PFOSA treatment, singly or in combination with CoCl_2_ or DFO, groups and control group was done using One-way ANOVA with post-hoc (Tukey) using SPSS. We used Simca-P 12 to perform multivariate analysis making principal component analysis (PCA) plots. All observations and variables of concern were investigated and based on distribution patterns and group formation we chose which groups to investigate further. Variables investigated here were Q-PCR data. Observations (exposure groups) must be independent when investigated using PCA, so data was separated on terms of exposure duration (24 and 48 h). PCA biplot presented herein were produced by first component (PC1) and second component (PC2) and percent of variation (R2X) is displayed for each plot.

## Results

### Evaluation of cell viability and validation of hypoxia exposure

**Figure 1:**
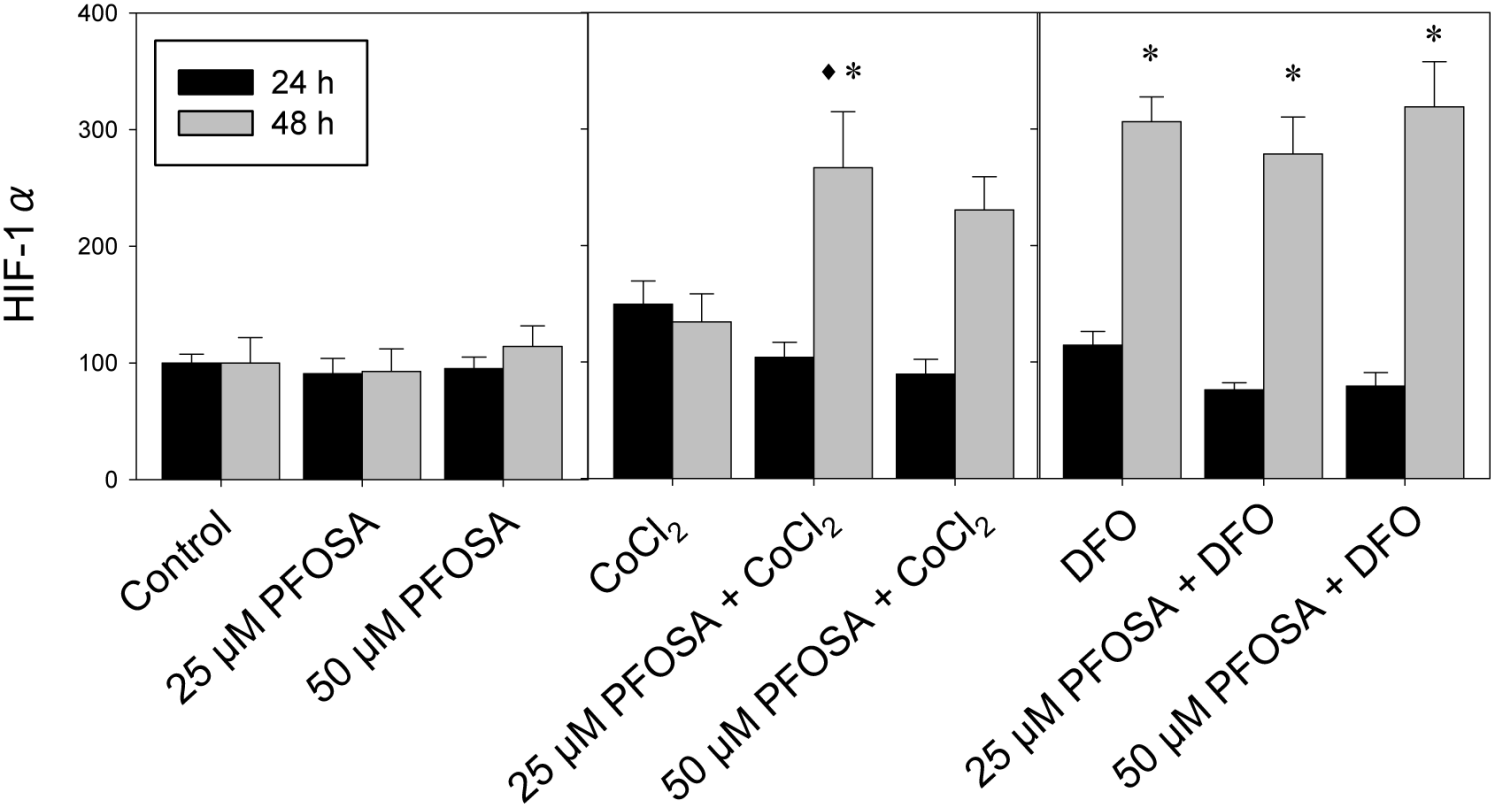
Changes in transcript levels for hypoxia-inducible factor 1α (HIF-1α) in salmon hepatocytes exposed to PFOSA (25 and 50 µM), singly or in combination with, either CoCl_2_ (150 µM) or DFO (100 µM) for 24 and 48 h. Transcripts were analyzed using real-time polymerase chain reaction (qPCR) and expressed as mean percentage (%) of control ± SEM (n = 5). Asterisk (*) denotes significant difference (p<0.05) compared to control analyzed by Tukey’s test, while diamond (^♦^) denotes significant difference (p<0.05) with individual hypoxia treatment group (CoCl_2_ or DFO) at respective time-interval.

Our pilot study showed that increasing DFO and CoCl_2_ concentration above 100 and 150 µM, respectively, noticeably reduced cell viability. Cell viability in the different exposure regimes showed a cell survival rate above 65% (data not shown). Gene expression analysis of HIF-1α was used to assess the hypoxic condition of the hepatocytes showing significant increase of mRNA expression, at 48 h compared to 24 h post-exposure, in combined exposure scenarios and by DFO alone. CoCl_2_ exposure induced HIF-1α after both 24 and 48 h, albeit not significant. HIF-1α mRNA was not induced by exposure to PFOSA alone (Fig. 1).

### Modulation of membrane FAs composition

Changes in membrane FA composition were observed after PFOSA, CoCl_2_ and DFO exposures and these effects were dependent on PFOSA concentration, combined exposure with individual DFO or CoCl_2_ and FA type (Table 2). After 24 h, exposure to DFO alone produced a significant reduction in ALA (18∶3n-3) levels, and combined low (25 µM) PFOSA in combination with CoCl_2_ or DFO increased ALA levels in salmon hepatocytes (Table 2). High PFOSA (50 µM) exposure, singly or in combination with CoCl_2_ or DFO, increased membrane ALA levels at 24 h exposure (albeit not significant). Exposure of hepatocytes to PFOSA or in combination with hypoxic condition significantly reduced membrane levels of ARA (20∶4n6) and EPA (20∶5n-3) after 24 h (Table 2). All exposure conditions produced increases in 22∶6n-3 (DHA) after 24 h. Linoleic acid (LA: 18∶2n-6) was increased in all exposure groups with CoCl_2_ and by 25 µM combined with DFO, while combined 50 µM PFOSA and DFO reduced LA level after 24 h (Table 2). Membrane ALA and GLA were reduced by DFO after 24 h, but were generally increased by other exposure regimes (Table 2). Membrane FA showed different composition pattern at 24 h, compared to 48 h (Table 2 and 3). At 48 h, ARA was significantly reduced in all exposure groups, except PFOSA that produced an increase. Membrane ARA levels were significantly reduced by CoCl_2_ and DFO exposure alone, compared with PFOSA exposure that increased ARA levels. Combined exposure with PFOSA and DFO or CoCl_2_ sustained the hypoxic condition mediated decrease on membrane ARA levels (Table 3). Membrane EPA levels were reduced by CoCl_2_ and DFO exposures at 48 h, and combined exposure with PFOSA concentrations significantly increased these effects, except combined DFO and 50 µM PFOSA, that produced a significant reduction (Table 3). While 25 µM PFOSA significantly reduced EPA levels, 50 µM PFOSA significantly increased membrane EPA levels after 48 h exposure (Table 3). Membrane LA levels were significantly increased by CoCl_2_ and DFO exposures alone or in combination with PFOSA concentrations at 48 h. On the other hand, membrane ALA and GLA were significantly reduced by CoCl_2_ and DFO exposure alone at 48 h, and combined exposure with 25 µM PFOSA significantly increased (DFO) and decreased (CoCl_2_) ALA levels, and the opposite is true for GLA at 48 h (Table 3). All exposure conditions reduced membrane DHA levels except combined DFO and 25 µM PFOSA, and combined CoCl_2_ and 50 µM PFOSA (Table 3).

**Table 2:** Fatty acid (FA) profile in primary culture of Atlantic salmon hepatocytes exposed for 24h to CoCl_2_ (150 µM) or DFO (100 µM), singly and in combination with PFOSA (25 and 50 µM).

After 24 hours of exposure										
Lipid		Control	25 uM PFOSA	50 uM PFOSA	150 uM CoCl2			100 uM DFO		
Name	Common name				No PFOSA	25 uM FPOSA	50 uM PFOSA	No PFOSA	25 uM FPOSA	50 uM PFOSA
14:0	Myristic Acid	100±12	315.4±46*	161.6±22*	391.3±81*	399.4±52*	246.5±29*	244.9±38*	223.8±55*	334.3±36*
14:1	Myrestoleic acid	100±8	**103.4±25**	258±24*	309.7±67*	355.8±29*	234.3±41*	148.6±33*	342.3±48*	236.3±38*
15:0	Pentadecylic acid	100±16	72.01±26*	***32.8*** **±** 11*	352.6±46*	**107.6±16**	*48.1*±14*	**120.7±14**	***53.3*** **±** 12*	149.1±32*
15:1	Pentadecenoic acid	100±22	209.1±43*	324.4±71*	311.8±28*	391.2±43*	217.2±46*	309.7±22*	376.2±26*	399.8±49*
16:0	Palmitic acid	100±9	75.7±12*	**103.4±26**	**114.2±15**	***11.8*** **±** 2*	*10.4*±3*	**114.3±15**	***45.1*** **±** 10*	**110.9±14**
16:1	Palmitoleic acid	100±21	158.2±42*	418.5±72*	349.4±42*	201.4±26*	376.4±44*	184±10*	230.2±15*	***52.3*** **±** 26*
17:0	Margaric acid	100±13	**80.9±21**	**97.3±31**	321.1±31*	**110.2±14**	*67.2*±18*	***53.3*** **±** 11*	281.3±28*	***44.6*** **±** 11*
17:1	heptadecenoic acid	100±21	12.9±2*	403.9±72*	293.3±35*	***43.5*** **±** 12*	99.5±15	***51.9*** **±** 9*	313.8±42*	***21.5*** **±** 4*
18:0	Stearic acid	100±7	**107.2±21**	***16.4*** **±** 2*	***14.7*** **±** 3*	***17.2*** **±** 7*	*13.9*±3*	255.8±21*	***16.4*** **±** 3*	***76.9*** **±** 15*
18:1n9c+18:1n9t	Oleic acid+Elaidic acid	100±11	***12.5*** **±** 4*	***28.3*** **±** 7*	***10.9*** **±** 2*	***31.5*** **±** 9*	*13.9*±2*	20.4±18*	***50.9*** **±** 12*	***12.4*** **±** 2*
18:2n6c+18:2n6t	Linolelaidic acid (LA)	100±13	**87.1±24**	**97.7±12**	181.3±22*	142.7±11*	165.6±16*	**84.3±14**	308.1±32*	***73.3*** **±** 11*
18:3n6	γ-linoleic acid (GLA)	100±26	**82.8±26**	**115.4±22**	**124.6±11**	160.7±23*	149.2±11*	***64.6*** **±** 19*	348.8±62*	***64.4*** **±** 12*
18:3n3	α-linolenic acid (ALA)	100±21	**113.4±26**	**127.2±17**	**97.3±15**	325.5±59*	151.1±21*	***12.4*** **±** 3*	454.2±38*	**123.5±14**
20:0	Arachidic acid	100±22	**123.7±26**	185.1±28*	**132.1±15**	**67.6±14**	***19.2*** **±** 12*	169.7±42*	**88.6±26**	**101.1±11**
20:1n9	Eicosenoic acid	100±31	163±41*	**117.3±16**	174.4±37*	245.6±29*	224.3±38*	208.4±32*	***13.3*** **±** 1*	153.4±14*
20:2	Eicosadienoic acid	100±11	***24.8*** **±** 3*	***54.2*** **±** 14*	**73.2±17**	***34.2*** **±** 15*	***15.6*** **±** 2*	***14.1*** **±** 2*	***55.2*** **±** 12*	***43.2*** **±** 9*
20:4n6	Arachidonic acid (ARA)	100±8	***48.1*** **±** 14*	***69.8*** **±** 11*	**80.5±21**	***65.9*** **±** 23*	***49.8*** **±** 14*	***65.6*** **±** 14*	***45.4*** **±** 11*	***74.2*** **±** 11*
20:5n3	Eicosapentaenoic acid (EPA)	100±22	***34.9*** **±** 9*	***30.5*** **±** 7*	**76.6±14**	***34.1*** **±** 14*	***24.5*** **±** 8*	***23.5*** **±** 3*	***39.2*** **±** 8*	***45.4*** **±** 12*
21:0	Heneicocylic acid	100±15	***66.7*** **±** 15*	206.8±31*	179.2±22*	212.8±16*	***50.9*** **±** 10*	**71.8±16**	***41.6*** **±** 11*	***63.7*** **±** 12*
22:0	Behenic acid	100±26	**94.8±28**	**73.6±32**	146.3±39*	**77.2±10**	***65.8*** **±** 12*	**132.3±132**	190.6±16*	**132.6±21**
22:1n9	Erucic acid	100±17	190.5±31*	**116.1±15**	**87.2±15**	247.3±57*	153.6±26*	**133.8±29**	172.6±31*	165.1±31*
22:2	Docosadienoic acid	100±16	156.1±41*	307.3±62*	224.6±51*	262.1±21*	308.3±15*	240.9±35*	***60.2*** **±** 21*	**131.9±36**
22:6n3	Docohexaenoic acid (DHA)	100±33	249.6±34*	340.8±27*	198.3±22*	331.2±45*	219.8±26*	**147.3±21**	328.8±34*	**121.7±16**
24:0	Lignoceric acid	100±28	***58.3*** **±** 13*	***56.1*** **±** 13*	**95.4±14**	220.4±27*	153.8±16*	155.6±14*	341.3±41*	***52.1*** **±** 16*

Data are presented as mean (n = 5) percentage of control ± (SEM). Asterisk (*) denotes exposure groups that are significantly different from control (p<0.05). FAs that were significantly increased are shown in **bold**, while FAs that were significantly reduced are shown in *italics*.

**Table 3:** Fatty acid (FA) profile in primary culture of Atlantic salmon hepatocytes exposed for 48h to CoCl_2_ (150 µM) or DFO (100 µM), singly and in combination with PFOSA (25 and 50 µM). Data are presented as mean (n = 5) percentage of control ± (SEM).

After 48 hours of exposure										
Lipid		Control	25 uM PFOSA	50 uM PFOSA	150 uM CoCl2			100 uM DFO		
Name	Common name				No PFOSA	25 uM FPOSA	50 uM PFOSA	No PFOSA	25 uM FPOSA	50 uM PFOSA
14:0	Myristic Acid	100±13	**150.7**±32*	**198.5**±25*	97.3±23	**289.3**±29*	**142.2**±16*	**156.7**±38*	**155.2**±17*	73.8±26
14:1	Myrestoleic acid	100±16	74.6±19	**214.2**±23*	99.6±22	*53.5*±16*	104.2±19	*32.7*±7*	147.2±31	*25.5*±5*
15:0	Pentadecylic acid	100±34	105.2±17	76.8±19	90.8±11	*25.1*±4*	*52.8*±10*	*62.8*±19*	*65.3*±9*	*5.2*±1*
15:1	Pentadecenoic acid	100±21	100.9±16	*367*±41*	**350.8**±48*	**266.1**±27*	**303.2**±22*	121.2±121	**364.8**±62*	**327.4**±37*
16:0	Palmitic acid	100±12	85.6±12	123.2±44	*17.7*±2*	*12.5*±2*	124.3±19	74.2±18	*57.1*±8*	**312.2**±42*
16:1	Palmitoleic acid	100±8	*51.6*±10*	*28.9*±7*	*34.8*±16*	*58.7*±19*	82.1±12	**196**±42*	**242.2**±43*	*12.1*±6*
17:0	Margaric acid	100±18	82.4±21	128.3±47	**226.9**±26*	*48.9*±11*	*19.2*±4*	*52.2*±9*	**282.1**±24*	*13.6*±4*
17:1	heptadecenoic acid	100±22	70.5±25	84.8±13	97.9±29	*32.5*±6*	*21.4*±3*	103.2±23	**325.8**±29*	125.0±19
18:0	Stearic acid	100±21	84.2±19	*23.3*±4*	109.1±38	109.2±29	132.2±17	*35.2*±9*	**182.3**±39*	**145.6**±26*
18:1n9c+18:1n9t	Oleic acid+Elaidic acid	100±31	*47.3*±8*	*49.6*±13*	99.1±12	*38.6*±8*	*12.1*±2*	*21.2*±5*	*62.9*±18*	*28.8*±6*
18:2n6c+18:2n6t	Linolelaidic acid (LA)	100±15	**311.7**±38*	**258.6**±36*	**269.1**±42*	**316.6**±55*	**177.6**±15*	**213.2**±25*	**273.7**±26*	125.3±26
18:3n6	γ-linoleic acid (GLA)	100±28	*62.6*±21*	*60.7*±12*	*39.5*±14*	**278**±36*	**194.4**±52*	*52.3*±15*	82.4±31	*38.4*±10*
18:3n3	α-linolenic acid (ALA)	100±17	110.5±26	**173.2**±31*	97.8±19	*36.2*±12*	**163.1**±18*	*11.2*±7*	**362.3**±53*	*30.5*±14*
20:0	Arachidic acid	100±13	**230.1**±53*	128.4±13	**130.1**±29*	*32*±5*	**269.4**±42*	**279.3**±13*	123.3±26	101.2±13
20:1n9	Eicosenoic acid	100±28	*80.1*±14*	*20.4*±6*	**163.6**±32*	**242.7**±15*	**236.3**±29*	121.6±19	**183.2**±19*	*72.4*±12*
20:2	Eicosadienoic acid	100±12	*58.5*±17*	**312.7**±18*	94.2±18	89.8±26	*63.2*±26*	*26.1*±5*	*31.2*±7*	*55.4*±17*
20:4n6	Arachidonic acid (ARA)	100±18	**230.5**±12*	132.1±39	*25.7*±6*	*27*±9*	*42.1*±12*	*51.2*±18*	*57.4*±13*	*22.0*±8*
20:5n3	Eicosapentaenoic acid (EPA)	100±8	*50.1*±18*	**207.4**±29*	72.3±19	**146.5**±13*	**182.3**±28*	*11.3*±1*	**273.2**±18*	*33.5*±10*
21:0	Heneicocylic acid	100±16	*44.7*±8*	*19.7*±3*	*20.9*±4*	**148.1**±19*	**177.7**±26*	*52.3*±8*	*53.6*±8*	*15.3*±3*
22:0	Behenic acid	100±29	*13.4*±2*	101.8±10	*19.3*±3*	**248.8**±23*	*34.2*±10*	*47.5*±5*	**202.6**±19*	*21.3*±6*
22:1n9	Erucic acid	100±22	*47.7*±15*	117.9±6	*12.3*±3*	**204.9**±33*	132.4±19	*22.1*±19*	**184.6**±18*	*19.3*±6*
22:2	Docosadienoic acid	100±16	*28.7*±5*	127.5±31	*53.9*±21*	*63.5*±13*	**320.3**±34*	87.4±26	*55.3*±5*	*13.7*±2*
22:6n3	Docohexaenoic acid (DHA)	100±12	*20*±4*	*50*±15*	*58.9*±8*	*43.5*±26*	**231.8**±41*	*31.7*±19*	**353.8**±36*	*15.3*±3*
24:0	Lignoceric acid	100±27	*36.6*±7*	125.2±17	*15.6*±2*	**231.9**±41*	**183.2**±23*	*54.3*±11*	**152.3**±23*	*35.5*±4*

Asterisk (*) denotes exposure groups that are significantly different from control (p<0.05). FAs that were significantly increased are shown in **bold**, while FAs that were significantly reduced are shown in *italics*.

### Modulation of transcripts involved in fatty acid metabolism

**Figure 2:**
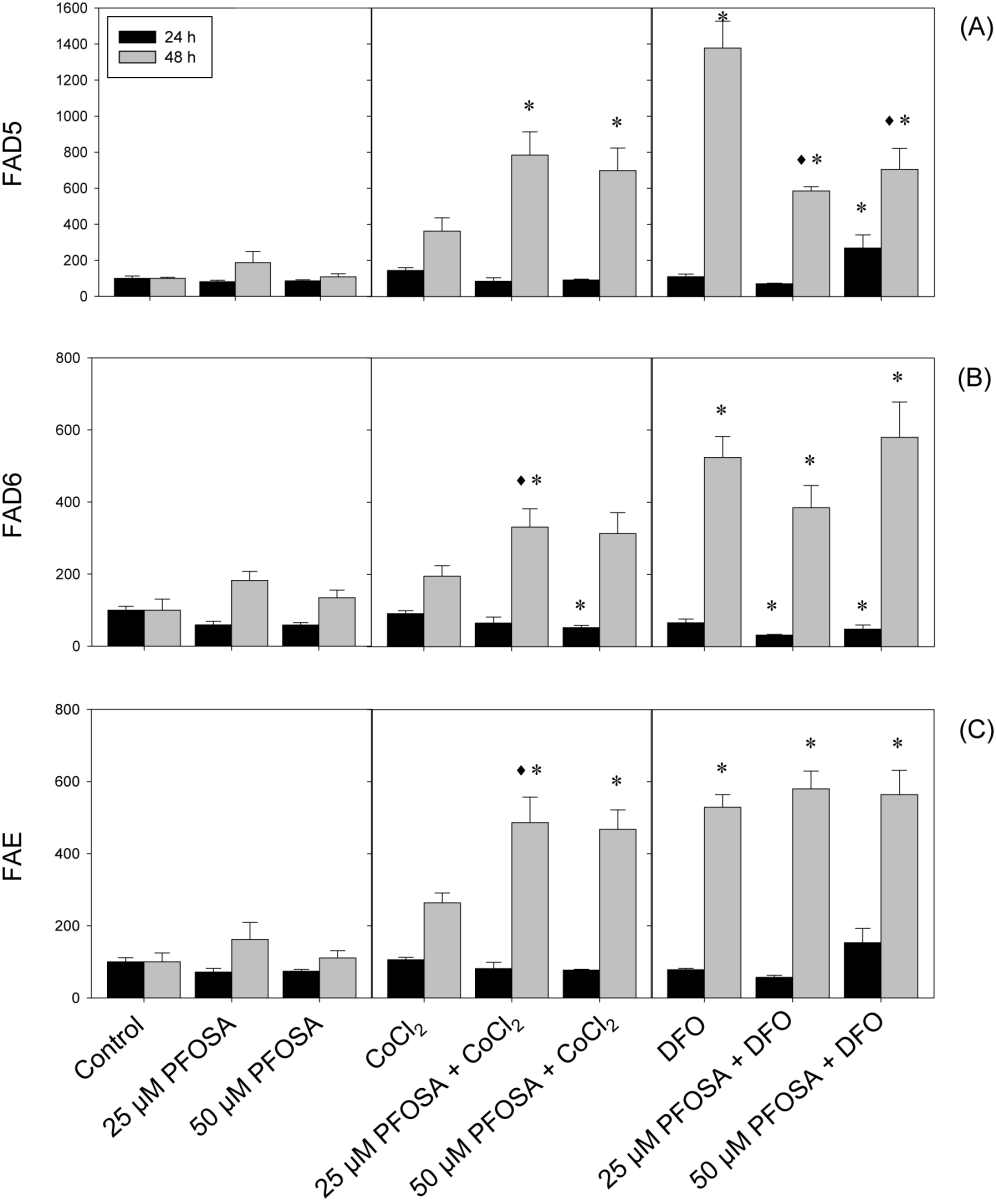
Modulation of FAD5 (A), FAD6 (B) and FAE (C) in salmon hepatocytes exposed to CoCl_2_ (150 µM) or DFO (100 µM), singly or in combination with PFOSA (25 and 50 µM). Transcripts were analyzed using real-time polymerase chain reaction (qPCR) and expressed as mean percentage (%) of control ± SEM (n = 5). Asterisk (*) denotes significant difference (p<0.05) compared to control analyzed by Tukey’s test, while diamond (^♦^) denotes significant difference (p<0.05) with individual hypoxia treatment group (CoCl_2_ or DFO) at respective time-interval.

**Figure 3:**
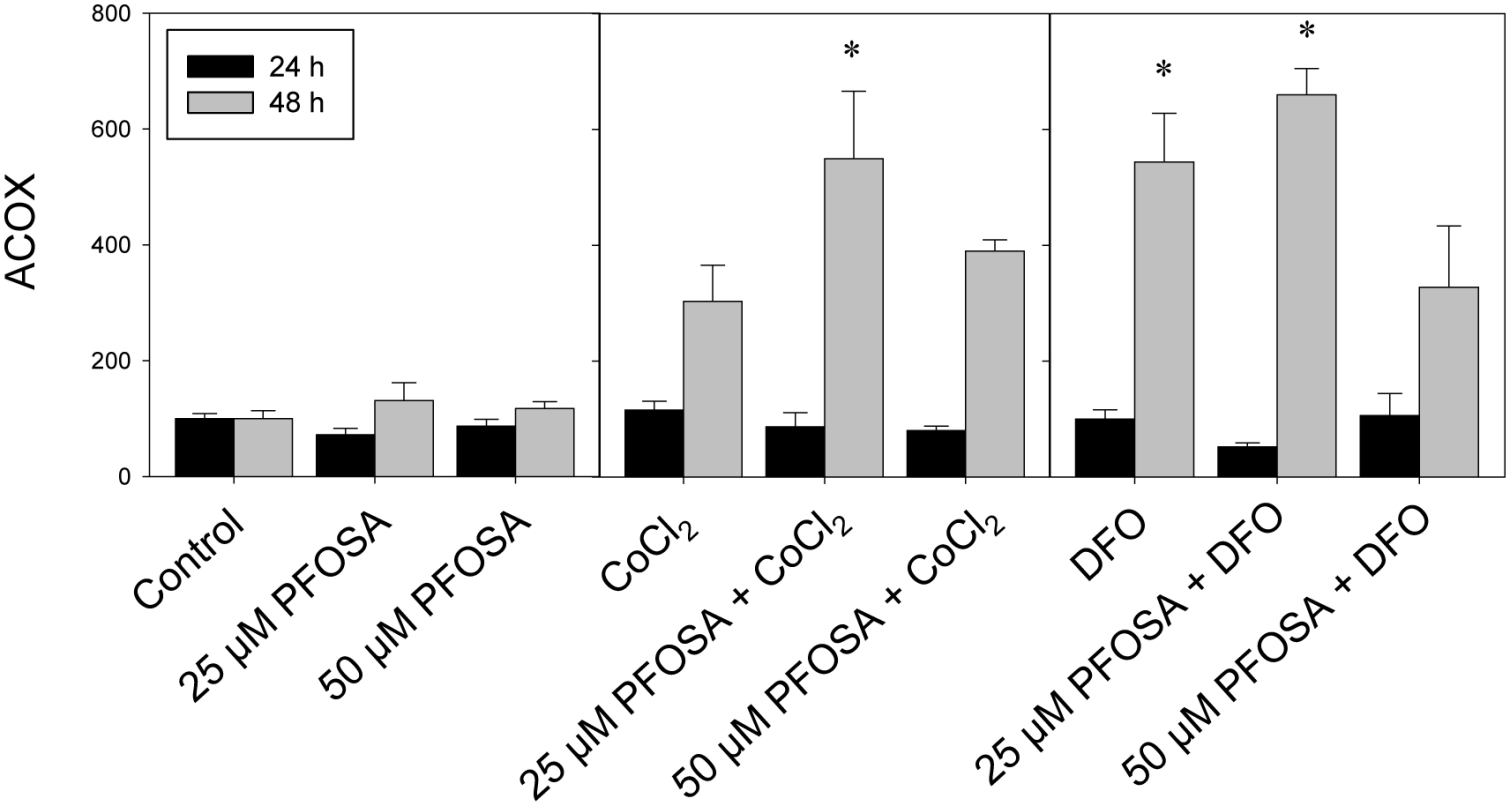
Transcriptional changes of ACOX mRNA in salmon hepatocytes exposed to CoCl_2_ (150 µM) or DFO (100 µM), in presence and absence of PFOSA (25 and 50 µM). Transcripts were analyzed using real-time polymerase chain reaction (qPCR) and expressed as mean percentage (%) of control ± SEM (n = 5). Asterisk (*) denotes significant difference (p<0.05) compared to control analyzed by Tukey’s test.

The effects of PFOSA, given singly or in combination with CoCl_2_ or DFO on FAD5, FAD6 and FAE, showed unique and comparable patterns after 24 and 48 h exposure (Fig. 2). Exposure to PFOSA concentrations increased transcription of FAD5, FAD6 and FAE mRNA at 48 h, while no significant effects were observed after 24 h (Fig. 2). The combined exposure of PFOSA and CoCl_2_ or DFO significantly increased FAD5, FAD6 and FAE transcripts at 48 h, while no significant effects were observed after 24 h exposure (Fig. 2A, B and C, respectively). Acyl-coenzyme A oxidase (ACOX) was not significantly affected by PFOSA exposure both at 24 and 48 h (Fig. 3). On the contrary, CoCl_2_ and DFO significantly increased ACOX mRNA expression at 48 h, and combined exposure with PFOSA concentrations significantly sustained these effects at the same time interval (Fig. 3). No effects were observed either when CoCl_2_ and DFO were given singly, or in combination with PFOSA concentrations at 24 h (Fig. 3).

### Modulation of transcripts involved in lipid peroxidation

**Figure 4:**
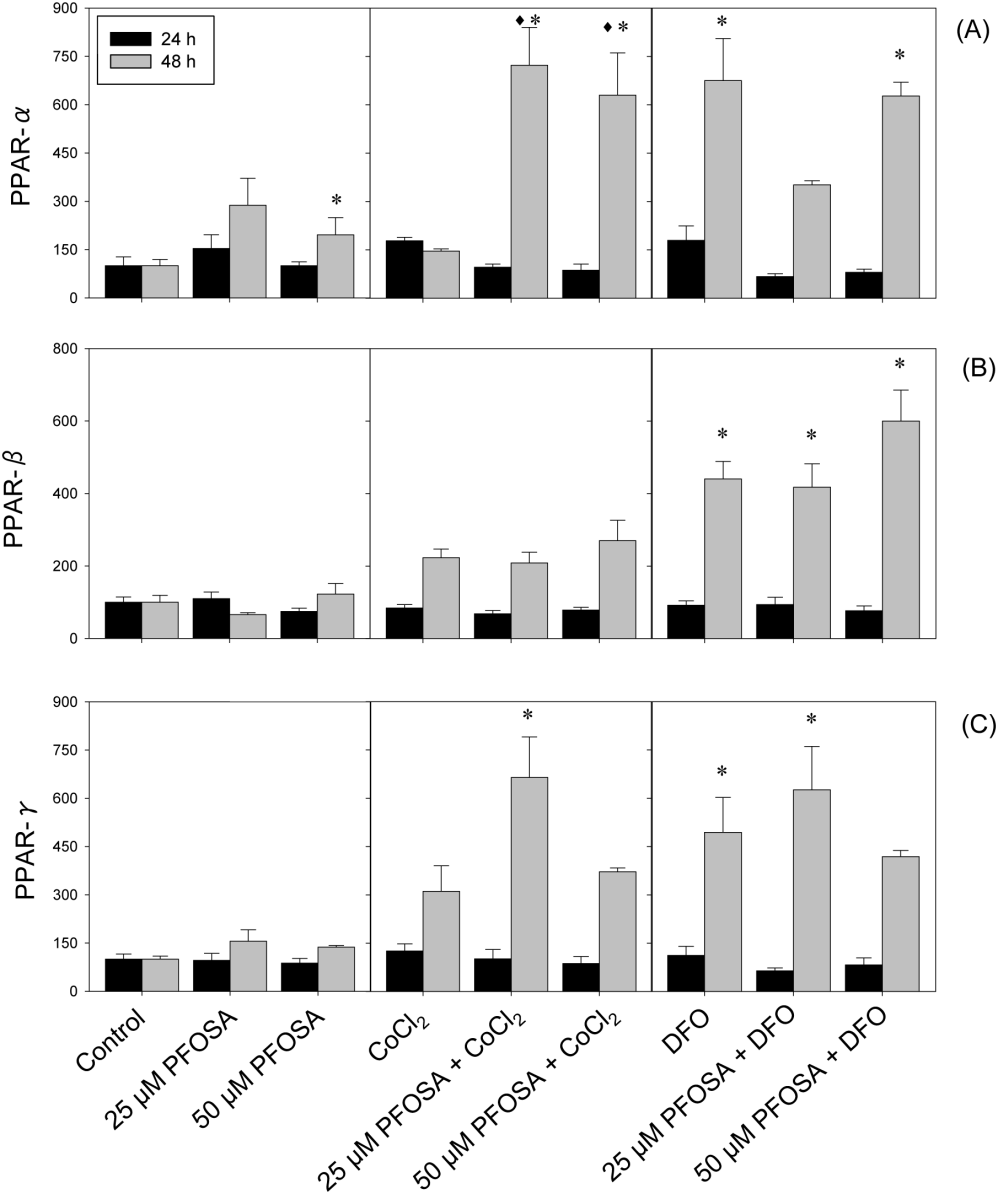
Modulation of PPAR-α (A), PPAR-β (B) and PPAR-γ (C) mRNA in salmon hepatocytes exposed to CoCl_2_ (150 µM) or DFO (100 µM), singly and in combination with PFOSA (25 and 50 µM). Transcripts were analyzed using real-time polymerase chain reaction (qPCR) and expressed as mean percentage (%) of control ± SEM (n = 5). Asterisk (*) denotes significant difference (p<0.05) compared to control analyzed by Tukey’s test, while diamond (^♦^) denotes significant difference (p<0.05) with individual hypoxia treatment group (CoCl_2_ or DFO) at respective time-interval.

Gene expression levels of PPAR (α, β and γ) were investigated in all exposure groups (Fig. 4) showing that exposure to PFOSA concentrations elevated PPARα mRNA levels at 48 h exposure (Fig. 4A). Exposure to CoCl_2_ alone did not affect PPARα, but combined exposure with PFOSA produced significant increase after 48 h exposure (Fig. 4A). On the other hand, exposure to DFO alone significantly increased PPARα mRNA at 48 h, and combined exposure with PFOSA concentrations sustained this effect, but with reduced expression levels in combination with 25 uM PFOSA (compared DFO exposure, Fig. 4A). For PPARβ and PPARγ, no effects were observed after exposure to PFOSA concentrations either at 24 or 48 h (Fig. 4B). On the other hand, CoCl_2_ and produced increases in PPARβ and PPARγ expressions, when given alone, and combined exposure with PFOSA concentrations significantly sustained these effects at 48 h (Fig. 4B and C, respectively). Otherwise, no significant PPARβ and PPARγ transcriptional changes were observed after 24 h exposure in any exposure group (Fig. 4B and C).

### Principal component analysis (PCA)

**Figure 5:**
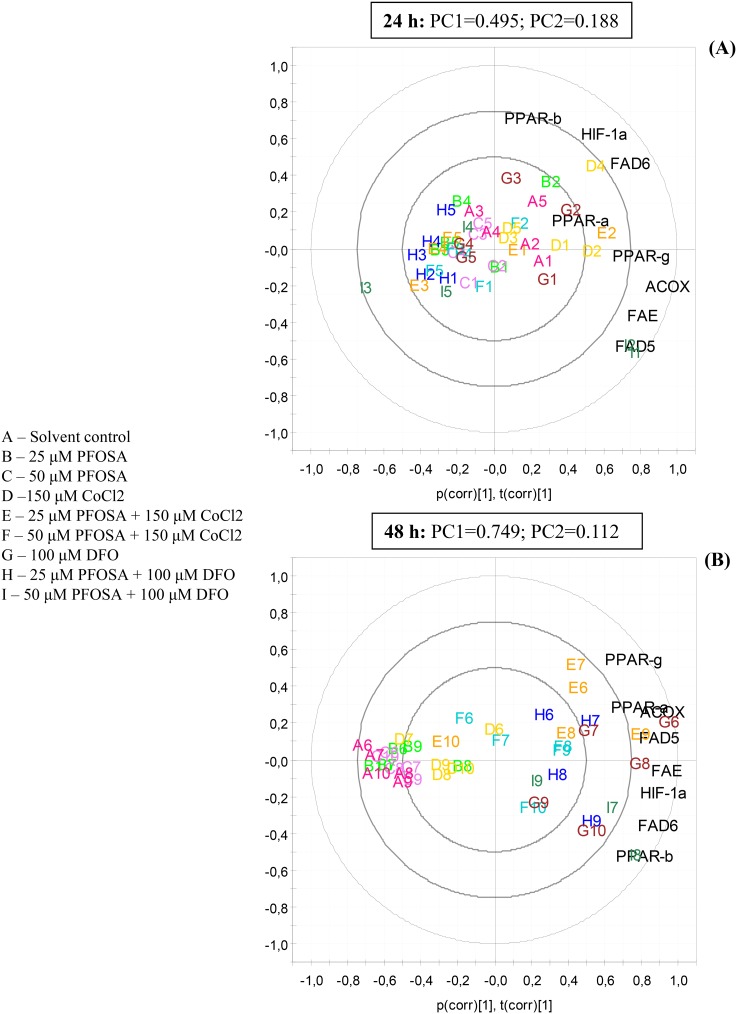
Biplot of principal component analysis (PCA) showing the scattering of HIF-1α, FAD5, FAD6, FAE, ACOX and PPAR (α, β and γ) mRNA levels after either 24 h (A) or 48 h (B) of exposure. Salmon hepatocytes were exposed to CoCl_2_ (150 µM) or DFO (100 µM) singly or in combination with PFOSA (25 and 50 µM) and gene expression was analyzed by qPCR. Letter denotes exposure treatment (A-Solvent control; B-25 µM PFOSA; C-50 µM PFOSA; D-50 µM CoCl2; E-25 µM PFOSA+150 µM CoCl2; F-50 µM PFOSA+150 µM CoCl2; G-100 µM DFO; H-25 µM PFOSA+100 µM DFO; I-50 µM PFOSA+100 µM DFO) and followed by a number (1–10) denoting the individual sample.

A principal component analysis was used in order to explore observations and variables with correlative patterns. We chose to incorporate molecular responses (mRNA) as variables and all comparable observations. At biplot analysis after 24 h exposure, we observed that all observations were located around neutral point (t[1] = 0, t[2] = 0) and there is no distinct distribution pattern among or between groups. Variables were located in the right side arc of the plot, and mostly explained by principal component 1 (PC1: 49.5%), except PPARβ that is located closer to PC2 (18.8%: Fig. 5A). There was no association between observations and variables, and further evaluation of variables was not pursued. Biplot of samples exposed for 48 h showed distribution along PC1 (74,9%), where observations were clustered, although somewhat overlapping, related to separate exposure treatments (Fig. 5B). PC1 (74.9%) explained most variation in this dataset and neither the observations nor parameters were drawn particularly to PC2 (11.2%). Control, single PFOSA (25 or 50 µM) and single 150 µM CoCl_2_ are located along PC1 and on the left side of PC2. Combined PFOSA (25 and 50 µM) and CoCl_2_ are located further right in biplot compared to control and 25 µM PFOSA group is generally located above PC1 towards PPAR (α and γ) and ACOX. Some individual exposures are less described by the model and are located close to neutral point. All groups containing DFO were located left of PC2, showing several observations that are close to PC1 or below (Fig. 5B). Variables that were distributed in an arc along the outer ring at right side of the biplot were mainly explained by PC1. In a longer distance away from PC1, were PPARs, with PPARγ and PPARα above PC1 and PPARβ below, while FAD5, FAD6, FAE, ACOX and HIF-1α are closer to PC1. Generally, PC2 describes very little of the variation in this biplot (Fig. 5B).

## Discussion

Previously, it has been shown in several studies that PFASs modulate the PPAR system and membrane FA homeostasis [38], and through these pathways induce peroxisome proliferation and oxidative stress responses [28], [38], [39]. Changes in the global climate are currently observed as increases in temperature and CO_2_ that subsequently produce reduction in oxygen partial pressure (pO2), and its availability to aquatic organisms. Oxygen is crucial for aerobic organisms that depend on it for cellular respiration, and because reduced environmental oxygen saturation (hypoxia) and environmental contaminants represent multiple environmental stressor. Hypoxia has been associated with effects on hormonal and biotransformation systems [40], [41], [42], and the relative importance of environmental hypoxia on organismal adaptive abilities responding to chemical insult are not well understood. Therefore, the present study was designed to investigate molecular and physiological effects of hypoxia and PFOSA, given singly and also in combination, on membrane FA composition and associated effects on molecular processes that regulate lipid homeostasis in fish, using a salmon hepatocyte *in vitro* model. Cellular hypoxia was induced using DFO and CoCl_2_, two chemicals that are frequently used to induce hypoxia in *in vitro* models, but also have an apoptotic potential [30].

### Modulation of membrane fatty acid composition

Hepatocytes adapt to reduction in oxygen levels by shifting energy production from mitochondrial fatty acid β-oxidation to glycolysis during periods of cellular hypoxia [43]. As a result, the activation of the HIF complex represents an early response to hypoxia exposure. Consequently, HIF is a central adaptive change in response to hypoxia through HIF-mediated reprogramming of cellular metabolism. Thus, HIF plays an integral role in switching energetic usage from aerobic to anaerobic metabolism to generate more ATP in an oxygen independent manner, through the regulation of glucose transporter 1 and several critical glycolytic enzymes, and to inhibit mitochondrial oxidative phosphorylation [44]. Glucose metabolism under hypoxic conditions and the role of HIF has been extensively studied, but less is known on its role in lipid metabolism in response to low oxygen and possible interaction with environmental contaminants. Recently, it was shown that acute and intermittent hypoxia induced liver lipid accumulation, suggesting a prominent role for HIF in regulating hepatic membrane lipid composition and metabolism [45], [46].

In the present study, we observed significant increase in transcript levels for HIF-1α mRNA expression after hypoxia (DFO and CoCl_2_) exposure, and this effect partially paralleled modifications in hepatic membrane FA composition, in the presence and absence of PFOSA. In addition, these effects did not parallel changes in transcript levels for FAD5, FAD6 and FAE. The relationship between increase in the composition of hepatic membrane FA composition and increase in FAD5, FAD6, FAE and HIF-1α expression is interesting amidst the ongoing controversy regarding the role of HIF-2 as a pro-lipogenic factor [47]. The finding showing that HIF-2α deficient mice exhibited hepatic steatosis, and the forced expression of hepatic HIF-1α, but not HIF-2α, that stimulated lipid accumulation in mice [48] suggests complicated roles of HIF-1α and HIF-2α in hepatic fat accumulation and possible maladaptive pathologies [43].

Previously, we showed that PFOSA produced time- and concentration-dependent alterations in the hepatic membrane content of several classes of FAs in salmon hepatocytes [39], [49], [50]. Herein, we show that exposure of cells to hypoxic conditions produced changes in hepatic membrane FA composition, similar to the effect of PFOSA alone, and combined exposure to hypoxia and PFOSA, further modulated the effects of hypoxic conditions alone. Note that these effects were based on hypoxia-inducing compound (DFO or CoCl_2_), exposure time and PFOSA concentration. PUFAs with 20 and 22 carbons are vital components of membrane phospholipids, and represent key steps in cell signalling, and control the expression of many genes involved in lipid synthesis and metabolism, thermogenesis, and cell differentiation [51]. For example, eicosanoids, including prostaglandins, thromboxanes and leukotrienes, belong to an extensive family of oxygenated metabolites derived from 20-carbon PUFAs such as ARA and EPA [52], which primarily act as potent local modulators in cells [53]. In accordance with previous findings [39], [49], [50] and as demonstrated in the present study, salmon liver is capable of FAD6-desaturation of ALA to stearidonic acid (18∶4n3) followed by elongation and FAD5 desaturation to EPA, in addition to FAD6 desaturation of Linoleic acid (18∶2n-6) to γ-linoleic acid (18∶3n6) followed by elongation to Dihomo-γ-linolenic acid (DGLA, 20∶3n-6) and FAD5 desaturation to ARA [54]. Our data show that hypoxic conditions reduced several n-3 PUFAs such as ALA, DHA and EPA, which were not in accordance with the increased expression of FAD5, FAD6 and FAE mRNA.

Furthermore, the availability of 20- and 22-carbon polyenoic FAs is highly dependent on the activity of FAD6, which mediates the rate-limiting step in the production of ARA [51]. The increase in FAD5, FAD6 and FAE mRNA expressions, three enzymes of the FA elongation pathway, paralleled the increase in membrane trans-linolelaidic acid (18∶2n-6t) and DHA levels (but not ARA). This discrepancy may be explained by the fact that in fish, n-3 PUFAs are abundant and play significant roles in immune function and apoptosis [55], [56]. In addition, the major regulation mechanism of FAD6 is assumed to be pre-translational [57], and our findings provide evidence that hypoxia increases the activity of the elongation machinery in order to adapt to membrane and physiological requirements due to the shortage of these FAs. Overall, the modulation of membrane FA composition observed in the present study predominantly involved an increase in FA methyl esters, indicating that hypoxia, given singly and also in combination with PFOSA, may affect lipid metabolism in Atlantic salmon. Over-production of ARA-derived eicosanoids may be responsible for a number of pathophysical conditions in humans, such as atherothrombotic and chronic inflammation diseases [58]. Thus, our data suggest that changes in membrane FA levels compensated for lipid peroxidation through an increase by elongation and desaturation activities in order to increase membrane fluidity as a form for compensatory mechanism. This speculation is supported by the fact that the β-oxidation pathway was positively enhanced in the hepatocytes after exposure to hypoxic conditions, singly and also in combination with PFOSA concentration (see below). Elsewhere, it has been shown that PFOSA, PFOA and PFOS reduced lipid synthesis and increase β-oxidation in rat *in vivo* system, with inconsistent changes in other enzymes involved in lipid metabolism [59]. The report showing that erucic acid inhibited peroxisomal β-oxidation in rats [60], provided strong support to our observed decrease of PUFAs and increase of mRNA levels for FA elongation enzymes and ACOX1 by hypoxic conditions, as it has been shown that PUFAs may repress peroxisomal β-oxidation [60]. Furthermore, it has been suggested that alteration of mRNA levels by FAs and PPAR activators is often disconnected [61]. For example, peroxisomal proliferators and essential FA deficient diets has been shown to elevate the mRNA levels for FAD5 and FAD6, while dietary PUFAs are known to repress these genes [57]. The peroxisomal proliferator, Wy14643, was also shown to produce delayed induction of FAD5 and FAD6 in rats, compared to the FA oxidation genes [57], prompting the authors to suggest that an induction of the desaturases could occur directly because of the degenerated direct repeat 1 (DR1) element that was reported in human FAD6 gene and binds PPARα [57].

In the present study, the hepatic membrane FA composition both decreased and decreased (depending on FA type, exposure condition and time) at 24 and 48 h, after exposure to hypoxic conditions, singly and also in combination with PFOSA concentration. Overall, the n-6:n-3 ratio was either slightly reduced (50% at 25 µM PFOSA, singly or in combination with CoCl_2_) or unchanged at 24 h exposure, while at 48 h exposure a respective 3.3- and 2.7-fold increase at 25 µM PFOSA singly or in combination with CoCl_2_, and respective 5.8- and 2.3-fold increase at DFO singly or in combination with 50 µM PFOSA, were observed. The observed selective hypoxia and PFOSA mediated increase in the n-6:n-3 PUFA ratio suggests a possible adaptive response towards acute hypoxic condition, representing a suggested mechanism for membrane defense against oxidative stress [62], [63] which we are currently investigating as well (Olufsen et al. in prep). Further on a mechanistic standpoint, whether the increase of the elongation enzyme genes that did not parallel decreases in certain PUFAs in salmon hepatocytes is a direct response of hypoxic conditions and PFOSA effects in activating PPARα, or a secondary effect that was derived from altered membrane FA patterns, remains to be elucidated. Regardless, these data provide significant overview on the physiological processes that are involved in the hepatic response to hypoxic stress, given singly or in combination with environmental contaminants, and emphasizes the potential negative impact of high lipid consumption on fish tolerance to environmental hypoxia [64].

### Modulation of peroxisome proliferation pathway

PPARs are important regulators of lipid and lipoprotein metabolism, glucose homeostasis, cellular differentiation and inflammatory responses [65], [66]. Therefore, any change in FA profile may have physiological consequences for normal membrane functioning [67]. Herein, we showed that hypoxia given singly or in combination with PFOSA produced an apparent time-dependent change in the transcriptional level of PPAR isoforms. It should also be noted that these transcriptional increases paralleled increases of HIF-1α, ACOX, FAD5, FAD6 and FAE mRNA in the combined hypoxia and PFOSA exposure groups. The relationships between these variables were also confirmed by the PCA showing clustering of combined exposure groups and distribution of samples after 48 h. Different distribution pattern at 24 and 48 h in the PCA bi-plot, implies that changes in mRNA responsiveness is time-dependent.

The role of PPARα in physiological processes such during angiogenesis has been investigated under hypoxia condition [68] and reviewed by [69], showing inductive and inhibitory effects [69]. For example, mitochondrial FA oxidative capacity was reduced by hypoxia, resulting in reduced mitochondrial lipid mobilization and utilization, and consequent accumulation of intracellular neutral lipid [70]. In another study, cardiomyocytes increased oxygen utilization efficiency by switching from FA oxidation to glycolysis under hypoxic conditions, and this shift of metabolic substrate was achieved by HIF-1-induced increase of the expression of glucose transporters and glycolytic enzymes [71], [72], and PPARα/RXR-mediated suppression of mitochondrial FA β-oxidation [70], [73]. Using two different *in vivo* systemic hypoxia models (CoCl_2_ and iso-volemic hemodilution), Razeghi and co-workers [74] reported a decrease in the expression of PPARα and several PPARα target genes including (pyruvate dehydrogenase kinase 4 (PDK4), muscle carnitine palmitoyltransferase-I (mCPT-I), and malonyl-CoA decarboxylase (MCD) in rat heart, and suggests a potential transcriptional mechanism for the decrease in long chain fatty acyl-CoA oxidation during hypoxia [74]. When the above mentioned reports are viewed with our data showing increased HIF-1α expression that paralleled PPAR isoforms, including PPARα – there are discrepancies as has been reported previously [69], regarding the cellular mechanism of peroxisomal β-oxidation towards hypoxia adaptation. It should be noted that we observed significant alterations of membrane FA profile towards hypoxia exposure. When the changes in membrane FA profile and PPARs data are taken together, there is a potential that hypoxia increased the level of endogenous ligands for all PPARs in salmon hepatocytes. This argument is supported by the observation showing no differences between PPAR isoforms, which were all increased by hypoxia exposure alone or in combination with PFOSA.

During normal physiological conditions, there is an inverse relationship between PPAR isoforms, where PPAR-α and PPAR-β show similar expression patterns [75], and share some endogenous ligands [17], [76], while PPAR-γ have a dissimilar function and other endogenous ligands [77]. In accordance with the present findings, hypoxia has previously shown to induce PPAR-γ expression [78]. While the mechanism for this effect is unclear, a possible mechanism to conserve energy during sub-optimal conditions was proposed [79]. Overall, while DFO induces hypoxia by chelating iron for excretion and subsequently reducing the potential for oxygen transport [29], CoCl_2_ is a transition metal that replaces iron in heme proteins, but does not bind oxygen, contrary to iron, when incorporated to protoporhyrins [80]. The entire iron replacement produces an oxygen sensor signal to the cell that mimics a state of oxygen reduction [74], [80]. The DFO and CoCl_2_ mechanisms induced hypoxia gene marker in hepatocytes and whether these represent a generalized mechanism in all cells remains to be investigated. However, other hypoxia parameters than HIF-1α, such as HIF-2α are responsible for PPAR regulation [81], that could further explain the changes in FA profile observed in the present study.

Regardless, we reported recently that PFOA, PFOS and PFOSA modulated lipid homeostasis and PPAR transcription in salmon *in vivo* and *in vitro* systems [39], [50]. It has also been suggested that certain POPs can interact with transcription factors in a similar manner as FAs, acting as a PPAR agonist [82]. Given that salmonid tissues are characterized by high concentrations of PUFAs, making them prone to oxidative damage [83] and fish are more protected from lipid peroxidation than mammals [83], the present data provide significant insight on the effects of hypoxia on cellular lipid homeostasis. Combined hypoxia and PFOSA exposures increased PPAR isoforms, suggesting that these emerging environmental stressors produced peroxisomal proliferation in salmon hepatocytes. Furthermore, PPARγ could be involved in the regulation of the peroxisomal β-oxidation pathway in Atlantic salmon [84], [85]. Long-chain FAs, which are exclusively metabolized in the peroxisomes, exert an inhibitory effect on the peroxisomal β-oxidation [60]. Therefore, the activation of the elongation pathway could therefore explain the increased expression of ACOX1 that was observed after exposure to combined hypoxia and PFOSA. ACOX catalyses the rate limiting-step in peroxisomal β-oxidation pathway of FA, and is commonly used as a biomarker for peroxisomal proliferation [86]. The role of PPARγ in fat accumulation, adipocyte differentiation and immune response, lipid and carbohydrate metabolism has been reported [87]. Particularly, an antagonistic interaction between PPARα and PPARγ in the maintenance of lipid homeostasis [88] has been suggested. Contrary to previous findings by Wågbø et al (2012) showing distinct and apparent concentration-dependent transcriptional increase of PPARγ by PFOSA exposure of salmon hepatocytes [85], the present study showed a comparable pattern of expression between PPARγ and PPARα after combined exposure to hypoxia and PFOSA.

Increased oxidative stress and lipid peroxidation in salmon fed a diet containing PFOS and PFOA was reported [49]. ROS accumulation is a potentially harmful outcome of systemic hypoxia [5], [89], [90], and increased peroxisome proliferation may worsen the situation by adding to ROS load [91]. Lipid peroxidation produces alteration in membrane lipid structure that may affect membrane lipids and change in functionality [91], [92]. Our data demonstrate increased PPAR transcription in combined hypoxia and PFOSA exposure, compared to single exposures, supporting the significance of multiple stressor investigations.

In summary, alteration of FAD5, FAD6 and FAE gene expression were generally more affected by hypoxia than PFOSA and combined exposure produced stronger effects than hypoxia alone. Regulation of lipid homeostasis is a very complex process with a myriad of pathways in the energetic budget and link to the immune system. Increased peroxisome proliferation may have detrimental effects due to alteration of lipid homeostasis and directly by increasing lipid peroxidation. Our data show that PPARs (α, β and γ) transcription were increased and these responses were stronger in hepatocytes experiencing combined hypoxia and PFOSA exposure. The combined effects of hypoxia and PFOSA on lipid homeostasis and β-oxidation in salmon hepatocytes suggest that these emerging multiple environmental stressors evoke deleterious effects with potential overt physiological consequences for development, reproduction and general health.
